# Supplementary material for: Molecular Docking Assessment of Cathinones as 5-HT2AR Ligands: Developing of Predictive Structure-Based Bioactive Conformations and Three-Dimensional Structure-Activity Relationships Models for Future Recognition of Abuse Drugs
Source: Molecules. 2023 Aug 24;28(17):6236. doi: 10.3390/molecules28176236 (PMC10488745; doi:10.3390/molecules28176236)
Supplement: Supplementary file 1 [file molecules-28-06236-s001.zip › molecules-2568348-supplementary.pdf]

Supplementary Materials

for

Molecular Docking Assessment of Cathinones as 5-HT$_{2A}$R Ligands: Developing of Predictive Structure-Based Bioactive Conformations and Three-Dimensional Structure-Activity Relationships Models for Future Recognition of Abuse Drugs

Contents

Table S1. Onsets and durations of actions of **SCs** 5-HT$_{2A}$R ligands compiling the TR.

Table S2. Structure-based alignment assessment of 5-HT$_{2A}$R ligands.

Table S3. Structure-based alignment assessment of 5-HT$_{2B}$R ligands.

Table S4. Best Open3DQSAR Models' (OH2 model at PC5) Predictive Abilities for TR.

Table S5. Best Open3DQSAR Models' (OH2 model at PC5) Predictive Abilities for TS.

Table S6. Best Open3DQSAR Models' (OH2 model at PC5) Predictive Abilities for TS$_{CRY}$.

Figure S1. The structure-based alignment within 5-HT$_{2A}$R-DPPC and OH2 probe-based *PLS-coefficients* (positive steric coefficients presented in green, negative steric coefficients depicted in yellow, positive electrostatic coefficients portrayed in red, negative electrostatic coefficients displayed in blue) for **23** (a, b); **11** (c, d); **19** (e, f). For the clarity of presentation, DPPC was omitted.

Figure S2. The structure-based alignment within 5-HT$_{2A}$R-DPPC and OH2 probe-based *PLS-coefficients* (positive steric coefficients presented in green, negative steric coefficients depicted in yellow, positive electrostatic coefficients portrayed in red, negative electrostatic coefficients displayed in blue) for **24** (a, b); **5** (c, d); **20** (e, f). For the clarity of presentation, DPPC was omitted.

Figure S3. The structure-based alignment within 5-HT$_{2A}$R-DPPC and OH2 probe-based *PLS-coefficients* (positive steric coefficients presented in green, negative steric coefficients depicted in yellow, positive electrostatic coefficients portrayed in red, negative electrostatic coefficients displayed in blue) for **12** (a, b); **21** (c, d); **6** (e, f). For the clarity of presentation, DPPC was omitted.

Figure S4. The structure-based alignment within 5-HT$_{2A}$R-DPPC and OH2 probe-based *PLS-coefficients* (positive steric coefficients presented in green, negative steric coefficients depicted in yellow, positive electrostatic coefficients portrayed in red, negative electrostatic coefficients displayed in blue) for **7** (a, b); **14** (c, d); **8** (e, f). For the clarity of presentation, DPPC was omitted.

Figure S5. The structure-based alignment within 5-HT$_{2A}$R-DPPC and OH2 probe-based *PLS-coefficients* (positive steric coefficients presented in green, negative steric coefficients depicted in yellow, positive electrostatic coefficients portrayed in red, negative electrostatic coefficients displayed in blue) for **3** (a, b); **22** (c, d); **10** (e, f). For the clarity of presentation, DPPC was omitted.

Figure S6. The structure-based alignment within 5-HT$_{2A}$R-DPPC and OH2 probe-based *PLS-coefficients* (positive steric coefficients presented in green, negative steric coefficients depicted in yellow, positive electrostatic coefficients portrayed in red, negative electrostatic coefficients displayed in blue) for **15** (a, b); **16** (c, d); **13** (e, f). For the clarity of presentation, DPPC was omitted.

Figure S7. The structure-based alignment within 5-HT$_{2A}$R-DPPC and OH2 probe-based *PLS-coefficients* (positive steric coefficients presented in green, negative steric coefficients depicted in yellow, positive electrostatic coefficients portrayed in red, negative electrostatic coefficients displayed in blue) for **28** (a, b); **29** (c, d); **30** (e, f). For the clarity of presentation, DPPC was omitted.

Figure S8. The structure-based alignment within 5-HT$_{2A}$R-DPPC and OH2 probe-based *PLS-coefficients* (positive steric coefficients presented in green, negative steric coefficients depicted in

yellow, positive electrostatic coefficients portrayed in red, negative electrostatic coefficients displayed in blue) for **31** (a, b); **32** (c, d); **33** (e, f). For the clarity of presentation, DPPC was omitted.

Figure 9. The structure-based alignment within 5-HT$_{2A}$R-DPPC and OH2 probe-based *PLS-coefficients* (positive steric coefficients presented in green, negative steric coefficients depicted in yellow, positive electrostatic coefficients portrayed in red, negative electrostatic coefficients displayed in blue) for **34** (a, b); **35** (c, d); **36** (e, f). For the clarity of presentation, DPPC was omitted.

Figure S10. The structure-based alignment within 5-HT$_{2A}$R-DPPC and OH2 probe-based *PLS-coefficients* (positive steric coefficients presented in green, negative steric coefficients depicted in yellow, positive electrostatic coefficients portrayed in red, negative electrostatic coefficients displayed in blue) for **37** (a, b); **38** (c, d); **39** (e, f). For the clarity of presentation, DPPC was omitted.

Figure S11. The structure-based alignment within 5-HT$_{2A}$R-DPPC and OH2 probe-based *PLS-coefficients* (positive steric coefficients presented in green, negative steric coefficients depicted in yellow, positive electrostatic coefficients portrayed in red, negative electrostatic coefficients displayed in blue) for **40** (a, b); **41** (c, d); **42** (e, f). For the clarity of presentation, DPPC was omitted.

Figure S12. The structure-based alignment within 5-HT$_{2A}$R-DPPC and OH2 probe-based *PLS-coefficients* (positive steric coefficients presented in green, negative steric coefficients depicted in yellow, positive electrostatic coefficients portrayed in red, negative electrostatic coefficients displayed in blue) for **43** (a, b); **44** (c, d). For the clarity of presentation, DPPC was omitted.

Figure S13. The structure-based alignment within 5-HT$_{2A}$R-DPPC and OH2 probe-based *PLS-coefficients* (positive steric coefficients presented in green, negative steric coefficients depicted in yellow, positive electrostatic coefficients portrayed in red, negative electrostatic coefficients displayed in blue) for **45** (a, b); **46** (c, d). For the clarity of presentation, DPPC was omitted.

Figure S14. The structure-based alignment within 5-HT$_{2A}$R-DPPC and OH2 probe-based *PLS-coefficients* (positive steric coefficients presented in green, negative steric coefficients depicted in yellow, positive electrostatic coefficients portrayed in red, negative electrostatic coefficients displayed in blue) for **6WGT** (a, b); **7WC6** (c, d); **7WC7** (e, f). For the clarity of presentation, DPPC was omitted.

Figure S15. The structure-based alignment within 5-HT$_{2A}$R-DPPC and OH2 probe-based PLS-coefficients (positive steric coefficients presented in green, negative steric coefficients depicted in yellow, positive electrostatic coefficients portrayed in red, negative electrostatic coefficients displayed in blue) for **6A93** (a, b); **7VOD** (c, d); **7VOE** (e, f). For the clarity of presentation, DPPC was omitted.

Figure S16. The structure-based alignment within 5-HT$_{2A}$R-DPPC and OH2 probe-based *PLS-coefficients* (positive steric coefficients presented in green, negative steric coefficients depicted in yellow, positive electrostatic coefficients portrayed in red, negative electrostatic coefficients displayed in blue) for **7WC4** (a, b); **7RAN** (c, d); **7WC5** (e, f). For the clarity of presentation, DPPC was omitted.

Figure S17. The structure-based alignment within 5-HT$_{2A}$R-DPPC and OH2 probe-based PLS-coefficients (positive steric coefficients presented in green, negative steric coefficients depicted in yellow, positive electrostatic coefficients portrayed in red, negative electrostatic coefficients displayed in blue) for **7WC9** (a, b); **7WC8** (c, d). For the clarity of presentation, DPPC was omitted.

Table S1. Onsets and durations of actions of **SCs** 5-HT$_{2A}$R ligands compiling the TR.

Name (Number)	OA	DA	Ref	Name (Number)	OA	DA	Ref
Cathinone and its derivatives				**Naphyrone (13)**	30 min	NA	[34]
**Cathinone (1)**	15-30 minutes	2–3 hours	[34]	**MDPV (14)**	intranasal administration: 30 minutes intravenous injection: 10–15 min	intranasal administration: 2–3 hours intravenous injection: 30 minutes	[34]
**Flephedrone (2)**	NA	NA	[34]	**Pyrovalerone (15)**	NA	NA	[34]
**Mephedrone (3)**	10-45 min	2–4 hours	[34]	**MDPPP (16)**	NA	NA	[21]
**Methcathinone (4)**	5 min	60-90 min	[34]	*Benzo[d][1,3]dioxole-based* ***SCs***			
**4-Bromomethcathinone (5)**	NA	NA	[21]	**MDBD (17)**	NA	NA	[34]
**3-Bromomethcathinone (6)**	NA	NA	[21]	**MDMA (18)**	30–45 min	4–6 hours	[34]
**2-Fluoromethcathinone (7)**	NA	NA	[21]	**Butylone (19)**	15-60 min	3-5 hours	[34]
**2-(Trifluoromethoxy)-methcathinone (8)**	NA	NA	[21]	**Ethylone (20)**	15 - 45 min	2 - 4 hours	[34]
Pyrovalerone-based ***SCs***				**MDEA (21)**	15-30 min	*Pyrovalerone-based* ***SCs***	
**α-PPP (9)**	NA	NA	[21]	**Methylone (22)**	15-45 minutes	2.5-4 hours	[34]
**4-Methyl-α-PPP (10)**	NA	NA	[21]	***SCs'*** *precursors*			
**4-Bromo-α-PPP (11)**	NA	NA	[21]	**Amphetamine (23)**	30-45 min	6-8 hours	[34]
**3-Bromo-α-PPP (12)**	NA	NA	[21]	**Methamphetamine (24)**	15-45 minutes	8-12 hours	[34]

[a]Onset of action; [b]Duration of action; [c]Not available.

Table S2. Structure-based alignment assessment of 5-HT_{2A}R ligands.

Code	AutoDock	Vina		SMINA		DOCK		PLANTS		Ref
SF[a]			vina	vinardo	ad4		chemplp	plp	plp95	
EC[b]					Experimental Conformation Re-Docking (ECRD)					
**6A93**	2.321[c]	1.785	1.436	1.673	2.624	2.321	1.153	1.923	1.457	[16]
**6A94**	1.456	1.432	0.981	1.975	1.524	1.456	1.465	1.573	1.451	[16]
**6WGT**	2.345	1.983	1.243	1.834	1.987	2.123	2.142	1.983	2.226	[56]
**6WH4**	2.563	1.783	1.783	1.832	2.552	2.463	2.453	2.672	2.823	[55]
**6WHA**	2.435	2.831	2.432	2.443	2.638	2.124	2.143	2.562	2.134	[55]
**7RAN**	2.641	1.964	2.162	1.847	1.487	2.362	1.965	1.257	2.364	[63]
**7VOD**	1.951	2.812	1.984	1.954	1.632	1.964	1.517	1.639	1.527	[64]
**7VOE**	2.632	1.924	2.226	2.624	2.247	2.481	2.624	2.514	1.987	[64]
**7WC4**	3.624	1.874	1.962	2.552	1.954	3.624	2.614	1.697	2.156	[61]
**7WC5**	3.228	2.632	1.625	2.326	2.658	3.514	1.847	1.992	2.634	[61]
**7WC6**	2.984	1.874	2.251	1.847	1.421	3.265	1.965	2.624	1.524	[61]
**7WC7**	2.632	1.695	1.984	2.625	2.624	2.659	1.748	2.748	1.695	[61]
**7WC8**	3.624	1.874	2.625	2.657	1.847	3.258	2.632	1.936	1.854	[61]
**7WC9**	2.998	1.994	1.889	1.842	2.624	3.627	1.951	1.874	1.958	[61]
**DA[d]**	42.85%	89.28%	75.00%	78.57%	77.14%	39.28%	78.57%	82.14%	78.57%	
					Randomized Conformation Re-Docking (RCRD)					
**6A93**	2.431	1.895	1.964	1.731	1.847	3.452	1.478	1.104	1.673	[16]
**6A94**	1.895	1.674	1.874	1.573	2.654	2.663	1.453	1.653	1.734	[16]
**6WGT**	2.342	2.016	1.342	1.994	1.548	2.461	2.432	2.342	2.445	[16]
**6WH4**	2.967	1.994	1.907	2.016	2.214	3.673	2.973	2.992	3.435	[55]
**6WHA**	3.123	2.932	2.695	2.774	2.418	2.452	2.645	2.234	2.893	[55]
**7RAN**	3.415	1.965	1.984	1.594	1.987	2.542	1.658	1.654	1.897	[63]
**7VOD**	2.967	1.864	2.564	2.654	1.574	2.641	1.777	1.874	1.574	[64]
**7VOE**	3.221	1.978	2.639	3.215	2.362	2.587	1.749	2.625	2.517	[64]
**7WC4**	3.264	2.154	2.478	2.514	1.547	2.624	2.514	1.787	2.657	[61]
**7WC5**	2.984	1.967	1.987	1.548	1.259	3.514	1.547	2.565	1.547	[61]
**7WC6**	3.624	1.784	1.874	2.147	2.254	1.625	1.638	2.565	2.699	[61]
**7WC7**	3.654	2.634	2.968	1.984	2.635	2.514	2.641	1.894	2.514	[61]
**7WC8**	2.471	1.894	2.987	1.874	2.514	2.657	2.848	1.847	1.784	[61]
**7WC9**	2.987	2.624	2.697	1.666	2.641	3.541	2.614	1.594	1.984	[61]
**DA**	32.14%	82.14%	75.00%	74.28%	71.43%	39.28%	75.00%	78.57%	71.43%	
					Experimental Conformation Cross-Docking (ECCD)					
**6A93**	2.563	1.953	2.321	1.863	2.415	2.431	2.541	2.564	2.451	[16]
**6A94**	2.553	2.483	2.973	2.653	2.639	3.543	2.314	2.774	1.996	[16]
**6WGT**	2.662	2.633	1.893	2.074	1.964	2.641	2.784	2.745	2.781	[16]
**6WH4**	2.931	1.973	1.963	1.943	2.984	2.896	2.741	2.553	2.963	[55]
**6WHA**	3.451	3.032	2.853	2.684	2.418	2.523	2.984	2.785	2.334	[55]
**7RAN**	2.148	1.987	1.847	2.964	2.954	2.965	2.641	2.657	2.651	[63]

**7VOD**	3.362	2.514	1.667	1.897	1.987	2.635	1.974	2.874	2.418	[64]
**7VOE**	2.547	1.874	1.984	2.364	3.165	2.558	2.419	2.457	2.984	[64]
**7WC4**	3.457	2.552	2.968	2.226	1.984	3.695	2.996	1.487	1.897	[61]
**7WC5**	2.547	2.639	2.687	1.987	1.874	2.487	2.874	2.695	2.635	[61]
**7WC6**	3.248	1.874	1.847	1.984	2.657	2.965	1.965	1.547	1.987	[61]
**7WC7**	3.451	1.698	2.625	2.635	2.996	2.952	2.624	2.652	2.635	[61]
**7WC8**	2.597	1.587	2.587	1.897	2.874	3.147	3.647	3.697	2.987	[61]
**7WC9**	2.544	1.848	2.647	2.631	2.985	3.894	3.447	4.235	2.634	[61]
**DA**	28.57%	75.00%	71.24%	71.24%	60.72%	35.71%	50.00%	50.00%	57.14%	

[a]Scoring function; [b]Experimental conformation; [c]Root-Mean-Square-Deviation measured between the heavy atoms of the ligand's experimental and the ligand's re-/cross-aligned conformation; [d]Docking accuracy.

Table S3. Structure-based alignment assessment of 5-HT$_{2B}$R ligands.

Code	AutoDock	Vina		SMINA		DOCK		PLANTS		Ref
**SF[a]**			**vina**	**vinardo**	**ad4**		**chemplp**	**plp**	**plp95**	
**EC[b]**				**Experimental Conformation Re-Docking (ECRD)**						
**4IB4**	2.542	1.238	1.431	1.563	2.634	4.312	3.342	2.213	3.431	[59]
**4NC3**	3.886[c]	1.101	1.235	1.243	2.625	3.453	2.864	2.223	2.835	[59]
**5TUD**	2.145	1.965	1.968	1.625	1.625	2.652	1.265	1.964	1.965	[57]
**5TVN**	2.563	1.214	1.452	1.896	1.564	2.412	1.563	2.233	3.231	[56]
**6DRX**	1.432	1.546	1.238	1.784	1.874	1.643	2.563	2.324	2.322	[60]
**6DRY**	2.541	1.426	1.316	1.353	1.236	1.321	1.356	1.452	2.112	[60]
**6DRZ**	1.735	1.745	1.452	1.584	2.624	1.678	2.346	2.467	1.254	[60]
**6DS0**	1.562	1.784	1.467	1.336	2.415	1.654	1.922	2.463	2.241	[60]
**7SQR**	2.642	1.524	1.625	1.458	1.632	1.652	1.625	1.847	1.842	[62]
**7SRR**	2.614	1.965	1.965	1.625	1.528	3.265	2.517	1.658	1.748	[62]
**7SRS**	2.642	1.897	1.457	1.254	1.985	2.514	1.695	1.695	1.666	[62]
**DA[d]**	64.28%	100.00%	100.00%	100.00%	81.81%	59.09%	72.72%	72.72%	69.64%	
				**Randomized Conformation Re-Docking (RCRD)**						
**4NC3**	4.436	1.998	2.431	2.443	1.625	4.312	2.952	2.474	2.231	[59]
**4IB4**	2.675	2.264	2.435	2.453	2.445	4.774	3.424	2.321	2.452	[76]
**5TUD**	2.658	1.284	1.958	1.632	2.639	2.642	1.542	1.254	2.651	[57]
**5TVN**	3.431	1.388	1.874	1.964	2.457	3.421	1.873	2.654	3.653	[56]
**6DRX**	1.763	1.974	2.374	1.904	1.635	1.214	2.342	2.436	2.563	[60]
**6DRY**	2.673	2.462	2.332	2.431	1.457	1.783	1.352	1.742	2.311	[60]
**6DRZ**	2.544	1.896	2.184	1.984	2.635	2.153	2.673	3.342	2.431	[60]
**6DS0**	1.573	1.783	1.974	2.203	2.547	2.142	2.374	2.835	2.341	[60]
**7SQR**	2.641	1.965	2.642	2.458	2.636	2.148	1.623	1.625	1.598	[62]
**7SRS**	2.524	1.784	2.437	1.451	1.598	1.845	2.526	2.545	1.784	[62]
**7SRR**	2.559	2.624	1.663	2.641	2.654	2.658	2.568	2.645	1.658	[62]
**DA**	50.00%	86.36%	68.18%	72.73%	68.18%	50.00%	63.64%	59.09%	54.55%	
				**Experimental Conformation Cross-Docking (ECCD)**						
**4NC3**	4.432	2.434	2.325	2.784	3.116	3.532	3.012	3.714	3.531	[59]
**4IB4**	2.778	2.874	1.924	2.984	2.453	4.321	4.321	2.777	4.683	[59]
**5TUD**	3.645	1.965	2.514	1.795	2.342	2.624	2.614	2.745	3.014	[57]
**5TVN**	4.321	1.984	1.963	1.943	1.324	4.352	2.346	2.796	4.546	[56]
**6DRX**	1.937	2.435	2.424	2.435	1.621	2.563	2.896	3.332	3.451	[60]
**6DRY**	2.456	1.974	2.894	2.984	2.434	2.523	2.324	2.431	2.345	[60]
**6DRZ**	2.563	2.332	2.425	2.452	2.002	2.452	2.346	2.467	1.254	[60]
**6DS0**	1.784	1.027	2.436	2.894	2.784	2.451	2.438	2.996	2.451	[60]
**7SQR**	2.654	2.515	2.415	2.514	2.983	2.624	3.265	2.963	2.584	[62]
**7SRS**	3.669	1.458	2.481	1.894	2.435	3.214	2.514	2.624	2.958	[62]
**7SRR**	3.337	2.147	2.659	2.584	3.113	4.621	2.514	3.624	2.774	[62]

**DA**	36.37%	72.73%	63.64%	54.55%	50.00%	27.27%	45.45%	36.36%	31.82%

[a]Scoring function; [b]Experimental conformation; [c]Root-Mean-Square-Deviation measured between the heavy atoms of the ligand's experimental and the ligand's re-/cross-aligned conformation; [d]Docking accuracy.

Table S4. Best Open3DQSAR Models' (OH2 model at PC5) Predictive Abilities for TR.

**Compound**	**p*K*i**	**Field/Cross-validation**								
		**STE**			**ELE**			**BOTH**		
		**LOO[b]**	**LSO[c]**	**AEP[d]**	**LOO[b]**	**LSO[c]**	**AEP[d]**	**LOO**	**LSO**	**AEP[d]**
**1**	6.00	5.12	4.62	1.13	5.65	5.24	0.56	5.53	4.95	0.76
**2**	6.00	4.73	5.45	0.91	4.86	5.14	1.00	4.60	5.13	1.14
**3**	5.68	4.78	5.24	0.67	4.49	4.97	0.95	4.64	4.94	0.89
**4**	5.23	5.12	4.23	0.73	4.99	4.35	0.56	5.03	4.78	0.33
**5**	5.20	5.34	6.12	0.78	5.72	5.36	0.34	5.49	5.12	0.19
**6**	5.00	5.67	4.75	0.46	4.92	4.57	0.26	5.08	4.36	0.36
**7**	5.00	5.18	5.12	0.40	4.96	4.46	0.29	4.94	5.35	0.21
**8**	5.00	5.35	4.45	0.45	4.96	5.13	0.09	4.94	4.24	0.41
**9**	5.60	5.12	4.33	0.88	5.50	5.18	0.26	5.35	4.95	0.45
**10**	5.50	5.12	6.71	0.80	5.43	5.56	0.06	5.46	5.71	0.13
**11**	5.40	4.91	4.35	0.87	4.91	5.13	0.38	4.99	4.76	0.53
**12**	5.00	4.96	5.57	0.63	4.91	5.46	0.28	5.04	5.21	0.13
**13**	4.96	4.72	4.86	0.37	4.78	4.93	0.11	4.94	5.12	0.09
**14**	4.88	4.94	5.63	0.61	4.86	5.11	0.13	4.76	4.99	0.12
**15**	4.88	4.87	5.35	0.61	4.87	4.45	0.22	4.98	5.55	0.39
**16**	4.20	5.42	5.52	1.27	5.06	5.35	1.01	5.01	4.77	0.69
**17**	5.20	5.67	5.32	0.45	5.73	5.13	0.30	6.02	5.67	0.65
**18**	5.11	4.84	5.31	0.54	5.06	4.78	0.19	4.86	5.02	0.17
**19**	4.88	4.61	5.34	0.37	4.85	4.36	0.28	4.88	5.12	0.12
**20**	4.88	5.01	6.42	0.99	5.11	5.26	0.31	4.95	5.13	0.16
**21**	4.88	5.52	5.35	0.56	5.26	5.79	0.65	5.15	4.97	0.18
**22**	4.88	5.52	5.69	0.73	5.05	5.27	0.28	5.16	4.78	0.19
**23**	4.88	5.54	5.24	0.51	4.92	4.35	0.29	5.04	5.36	0.32
**24**	4.88	5.03	5.66	0.47	5.44	5.14	0.41	5.42	5.12	0.39

[a]Predictions were obtained with FFD optimized models. [b]Leave-one-out cross-validation. [c]Leave-some-out cross-validation with 5-random-groups-out. [d]The absolute error of prediction of LOO and LSO cross-validations.

Table S5. Best Open3DQSAR Models' (OH2 model at PC5) Predictive Abilities for TS.

Compound	pK_i	Field/Cross-validation								
		STE			ELE			BOTH		
		LOO[b]	LSO[c]	AEP[d]	LOO[b]	LSO[c]	AEP[d]	LOO	LSO	AEP[d]
**25**	8.57	6.87	6.12	2.08	6.63	6.22	2.15	8.43	5.12	1.80
**26**	7.20	6.34	6.26	0.90	6.34	6.12	0.97	7.36	6.61	0.38
**27**	8.39	8.05	7.62	0.56	7.92	7.41	0.73	7.76	8.24	0.39
**28**	8.22	7.87	7.31	0.63	7.66	7.24	0.77	7.43	8.07	0.47
**29**	9.67	7.12	6.12	3.05	7.12	6.55	2.84	7.84	7.43	2.04
**30**	6.95	6.14	6.07	0.85	6.05	5.43	1.21	7.22	6.27	0.48
**31**	7.54	7.95	7.12	0.42	7.13	6.06	0.95	6.51	5.75	1.41
**32**	7.34	8.13	7.56	0.51	6.66	6.41	0.81	6.97	6.37	0.67
**33**	8.15	7.32	7.23	0.88	7.46	7.23	0.81	8.12	7.99	0.10
**34**	7.78	8.31	7.56	0.38	7.13	6.87	0.78	7.24	7.61	0.36
**35**	6.20	5.54	5.42	0.72	4.76	4.34	1.65	6.39	4.13	1.13
**36**	6.82	7.53	7.18	0.54	6.31	5.94	0.70	6.23	6.65	0.38
**37**	8.88	6.14	6.99	2.32	7.36	7.31	1.55	7.68	7.94	1.07
**38**	6.81	5.98	5.83	0.90	4.36	4.02	2.62	5.13	4.54	1.98
**39**	7.44	6.57	6.51	0.90	7.14	6.71	0.52	7.22	7.38	0.14
**40**	9.69	8.74	8.88	0.88	8.76	8.32	1.15	8.76	6.94	1.84
**41**	6.03	5.68	5.53	0.43	5.85	5.17	0.52	5.26	6.14	0.44
**42**	8.52	8.63	8.34	0.15	7.76	7.61	0.84	8.12	6.33	1.30
**43**	5.00	4.06	3.99	0.98	4.21	4.00	0.90	4.04	3.36	1.30
**44**	5.64	4.79	4.71	0.89	5.21	4.79	0.64	5.14	3.96	1.09
**45**	6.07	5.13	4.87	1.07	4.07	3.22	2.43	5.67	4.26	1.11
**46**	7.40	6.52	6.46	0.91	6.54	6.51	0.88	6.65	5.06	1.55

[a]Predictions were obtained with FFD-optimized models. [b]Leave-one-out cross-validation. [c]Leave-some-out cross-validation with 5-random-groups-out. [d]The absolute error of prediction of LOO and LSO cross-validations.

Table S6. Best Open3DQSAR Models' (OH2 model at PC5) Predictive Abilities for TS_{CRY}.

Compound	pK_i	Field/Cross-validation								
		STE			ELE			BOTH		
		LOO[b]	LSO[c]	AEP[d]	LOO[b]	LSO[c]	AEP[d]	LOO	LSO	AEP[d]
**6A93**	8.16	9.41	9.34	1.22	9.35	9.06	1.05	9.96	9.63	1.64
**6A94**	7.40	8.34	6.12	1.11	8.85	6.06	1.40	8.12	6.54	0.79
**6WGT**	8.63	7.76	6.43	1.54	7.45	7.26	1.28	7.52	7.12	1.31
**6WH4**	9.70	8.32	8.11	1.49	8.36	8.24	1.40	8.75	8.54	1.06
**6WHA**	9.08	10.43	10.23	1.25	10.87	10.15	1.43	10.13	10.77	1.37
**7RAN**	NA[e]	8.65	7.42	NA	8.65	7.18	NA	8.31	7.89	NA
**7VOD**	7.73	6.75	5.22	1.75	6.87	5.56	1.52	6.97	5.85	1.32
**7VOE**	8.47	10.64	9.61	1.66	10.88	10.14	2.04	10.12	10.26	1.72
**7WC4**	7.49	5.76	4.59	2.32	5.95	5.53	1.75	5.24	5.02	2.36
**7WC5**	NA	6.32	5.11	NA	6.23	5.12	NA	5.87	5.46	NA
**7WC6**	8.63	7.83	6.32	1.56	7.98	7.45	0.92	7.46	7.12	1.34
**7WC7**	8.55	7.54	7.32	1.12	7.75	7.62	0.87	7.34	7.09	1.34
**7WC8**	9.27	8.54	7.64	1.18	8.92	8.12	0.75	8.14	8.03	1.19
**7WC9**	NA	6.65	7.36	NA	6.65	8.47	NA	6.28	8.28	NA

[a]Predictions were obtained with FFD-optimized models. [b]Leave-one-out cross-validation. [c]Leave-some-out cross-validation with 5-random-groups-out. [d]The absolute error of prediction of LOO and LSO cross-validations. [e]Not available.

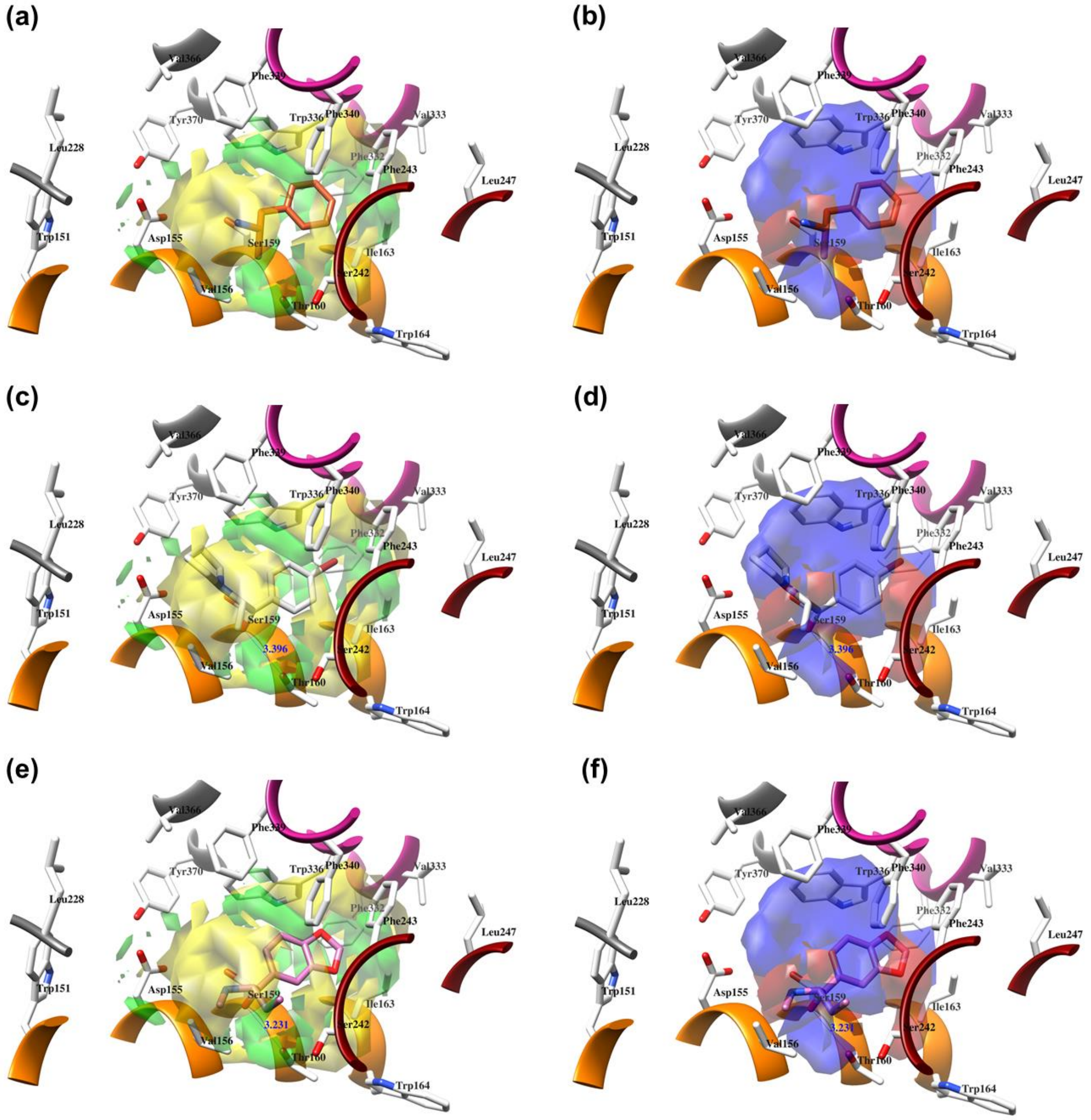

Figure S1. The structure-based alignment within 5-HT2AR-DPPC and OH2 probe-based *PLS-coefficients* (positive steric coefficients presented in green, negative steric coefficients depicted in yellow, positive electrostatic coefficients portrayed in red, negative electrostatic coefficients displayed in blue) for **23** (a, b); **11** (c, d); **19** (e, f). For the clarity of presentation, DPPC was omitted.

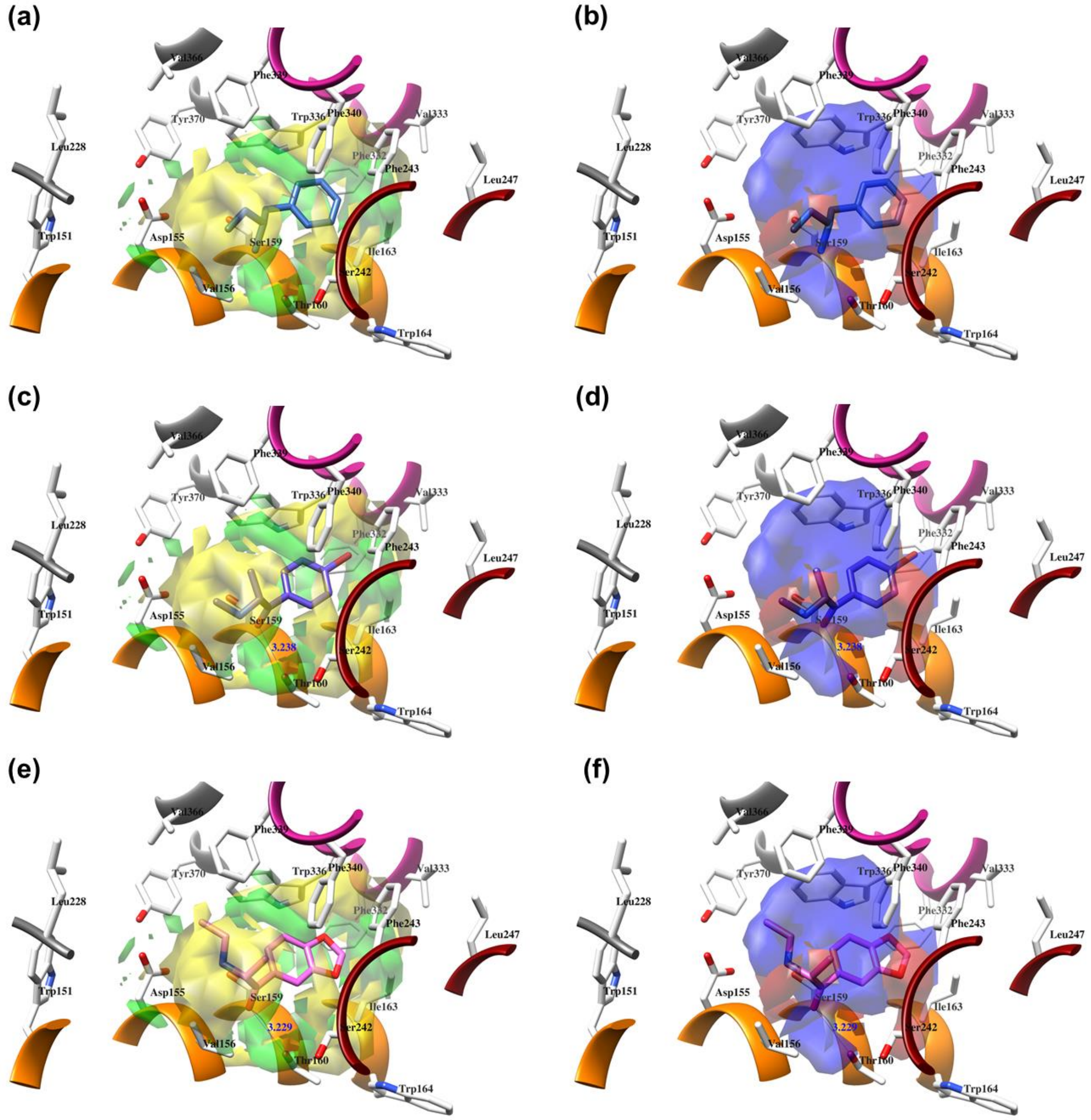

Figure S2. The structure-based alignment within 5-HT2AR-DPPC and OH2 probe-based *PLS-coefficients* (positive steric coefficients presented in green, negative steric coefficients depicted in yellow, positive electrostatic coefficients portrayed in red, negative electrostatic coefficients displayed in blue) for **24** (a, b); **5** (c, d); **20** (e, f). For the clarity of presentation, DPPC was omitted.

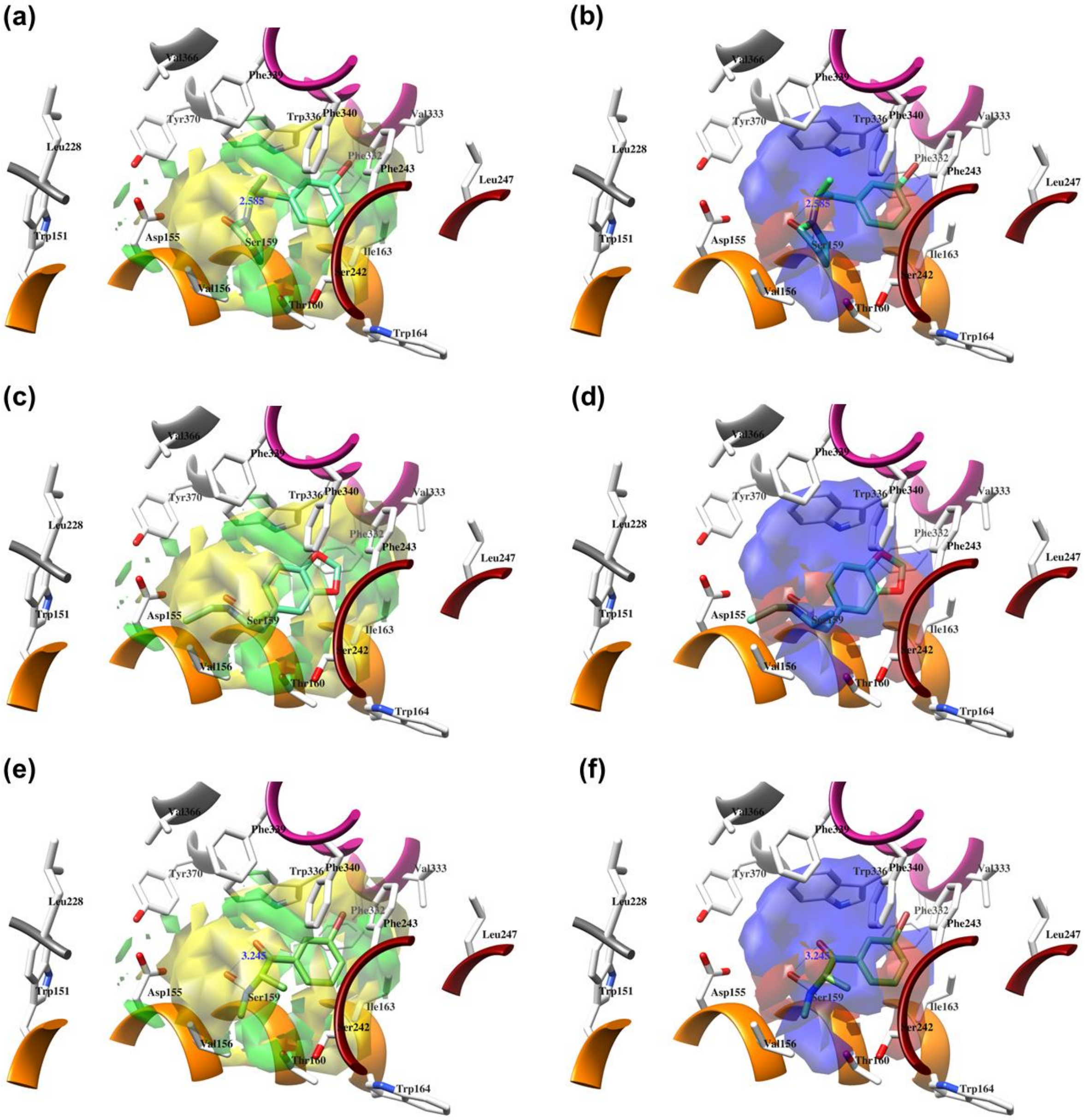

Figure S3. The structure-based alignment within 5-HT2AR-DPPC and OH2 probe-based *PLS-coefficients* (positive steric coefficients presented in green, negative steric coefficients depicted in yellow, positive electrostatic coefficients portrayed in red, negative electrostatic coefficients displayed in blue) for **12** (a, b); **21** (c, d); **6** (e, f). For the clarity of presentation, DPPC was omitted.

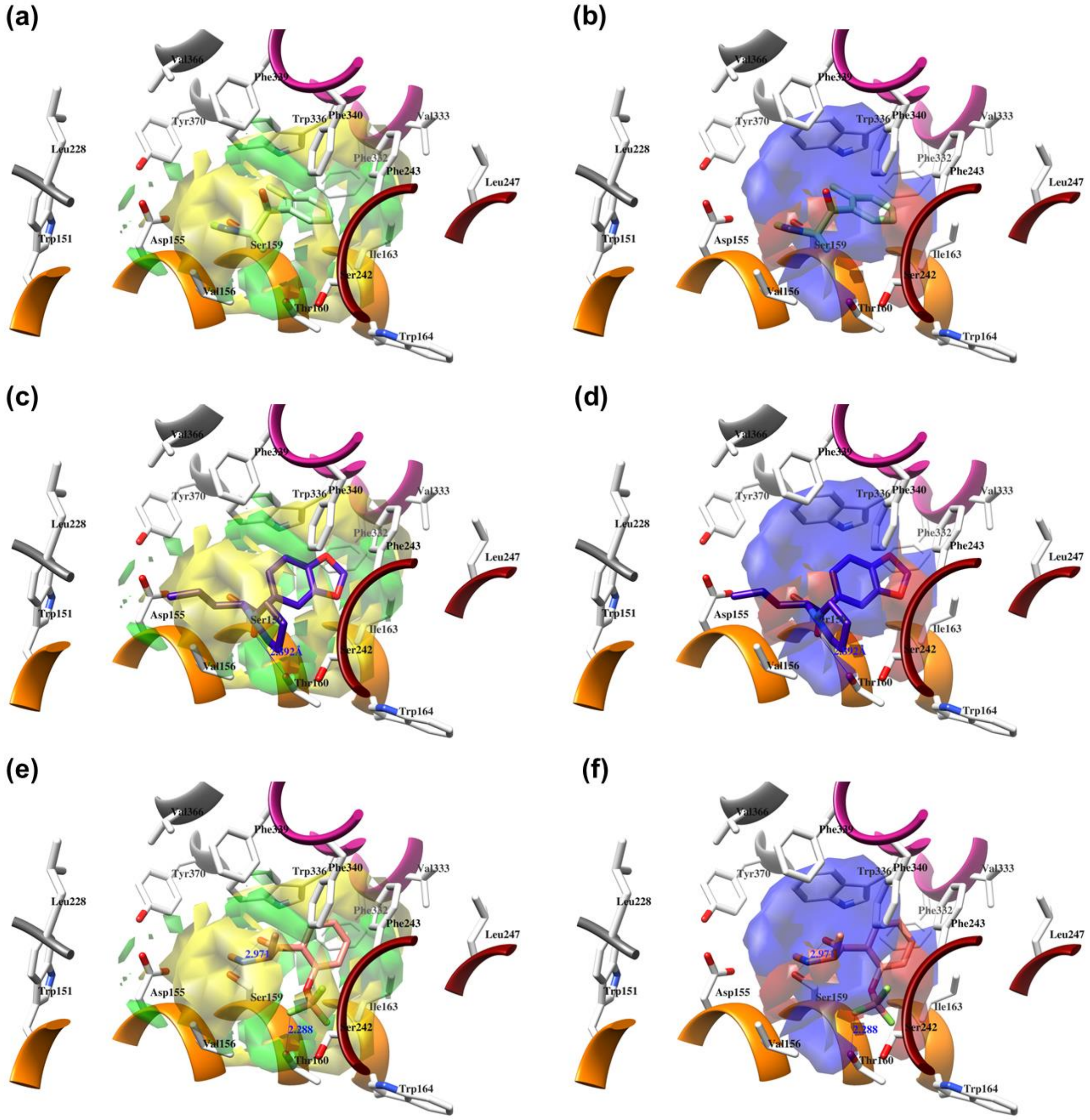

Figure S4. The structure-based alignment within 5-HT_{2A}R-DPPC and OH2 probe-based *PLS-coefficients* (positive steric coefficients presented in green, negative steric coefficients depicted in yellow, positive electrostatic coefficients portrayed in red, negative electrostatic coefficients displayed in blue) for **7**(a, b); **14** (c, d); **8** (e, f). For the clarity of presentation, DPPC was omitted.

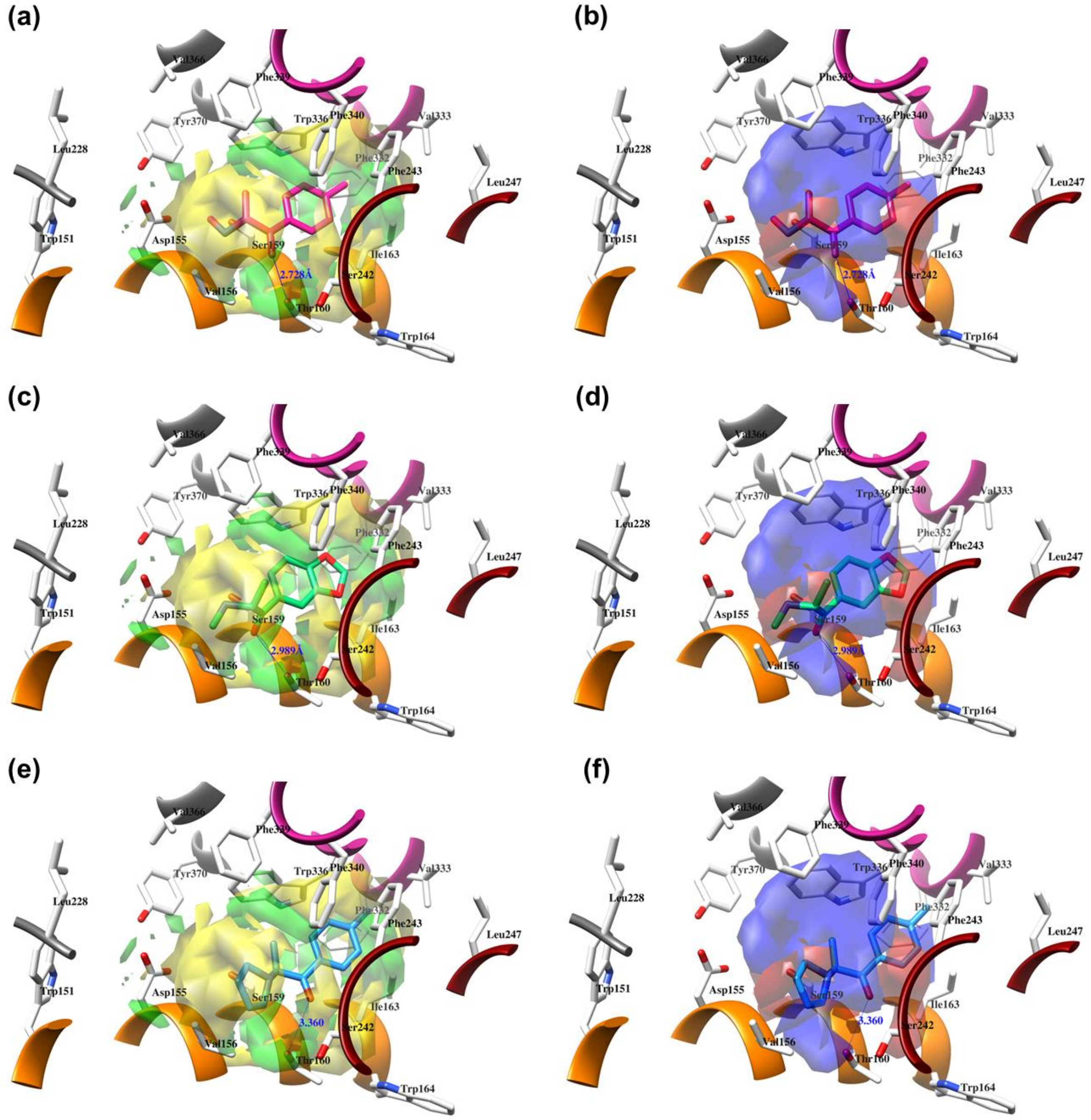

Figure S5. The structure-based alignment within 5-HT$_{2A}$R-DPPC and OH2 probe-based *PLS-coefficients* (positive steric coefficients presented in green, negative steric coefficients depicted in yellow, positive electrostatic coefficients portrayed in red, negative electrostatic coefficients displayed in blue) for **3** (a, b); **22** (c, d); **10** (e, f). For the clarity of presentation, DPPC was omitted.

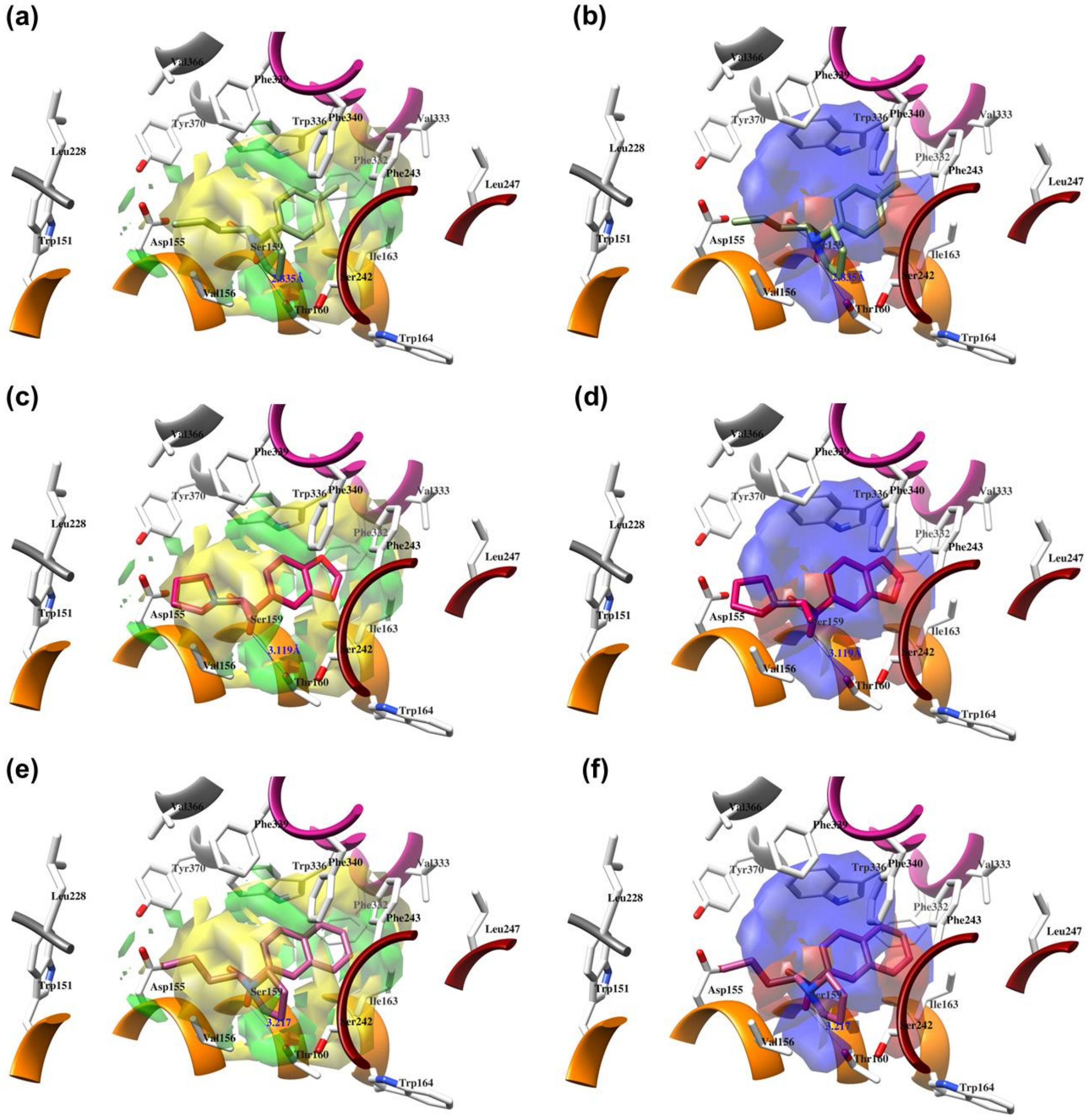

Figure S6. The structure-based alignment within 5-HT$_{2A}$R-DPPC and OH2 probe-based *PLS-coefficients* (positive steric coefficients presented in green, negative steric coefficients depicted in yellow, positive electrostatic coefficients portrayed in red, negative electrostatic coefficients displayed in blue) for **15** (a, b); **16** (c, d); **13**(e, f). For the clarity of presentation, DPPC was omitted.

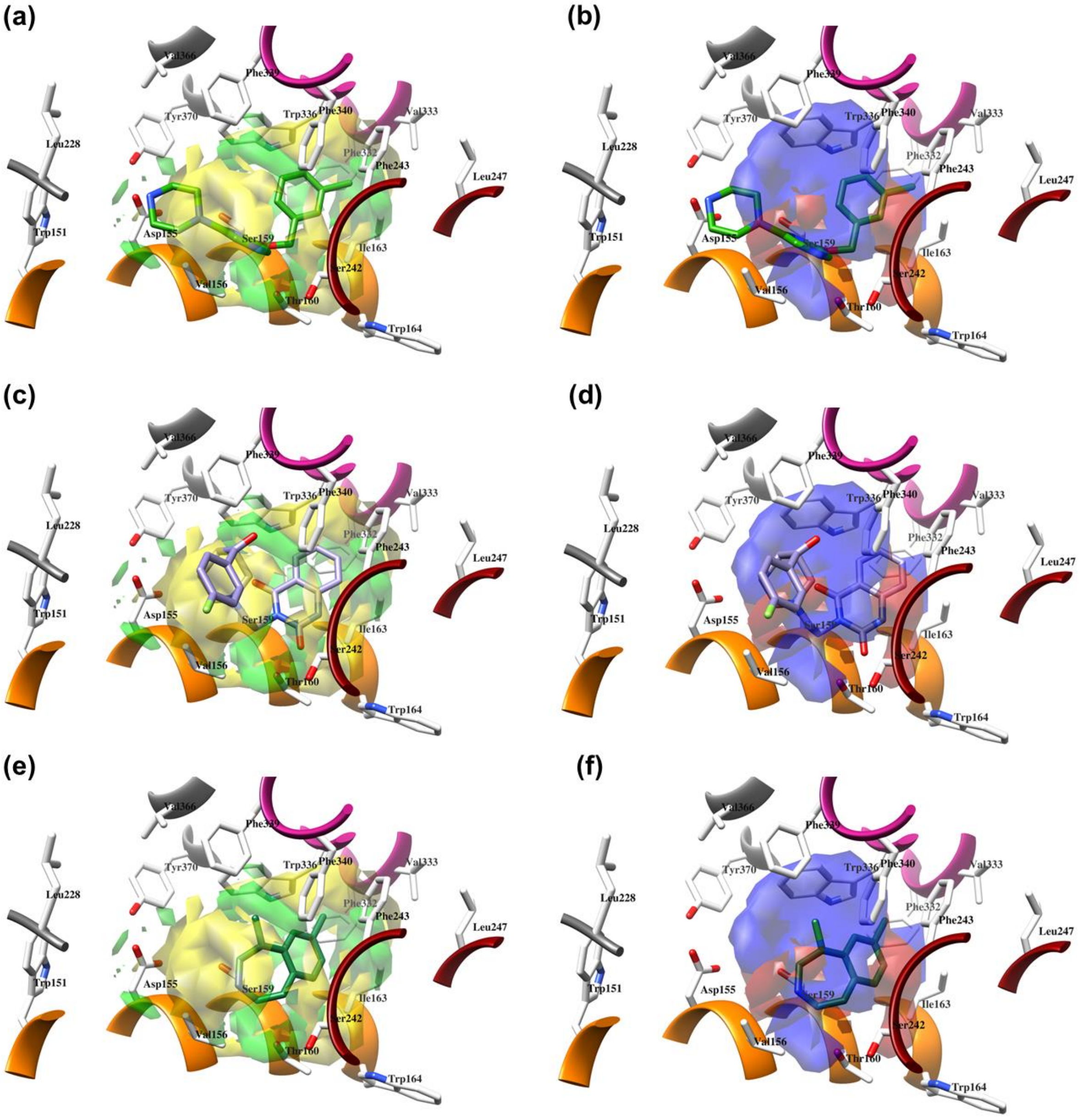

Figure S7. The structure-based alignment within 5-HT$_{2A}$R-DPPC and OH2 probe-based *PLS-coefficients* (positive steric coefficients presented in green, negative steric coefficients depicted in yellow, positive electrostatic coefficients portrayed in red, negative electrostatic coefficients displayed in blue) for **28** (a, b); **29** (c, d); **30** (e, f). For the clarity of presentation, DPPC was omitted.

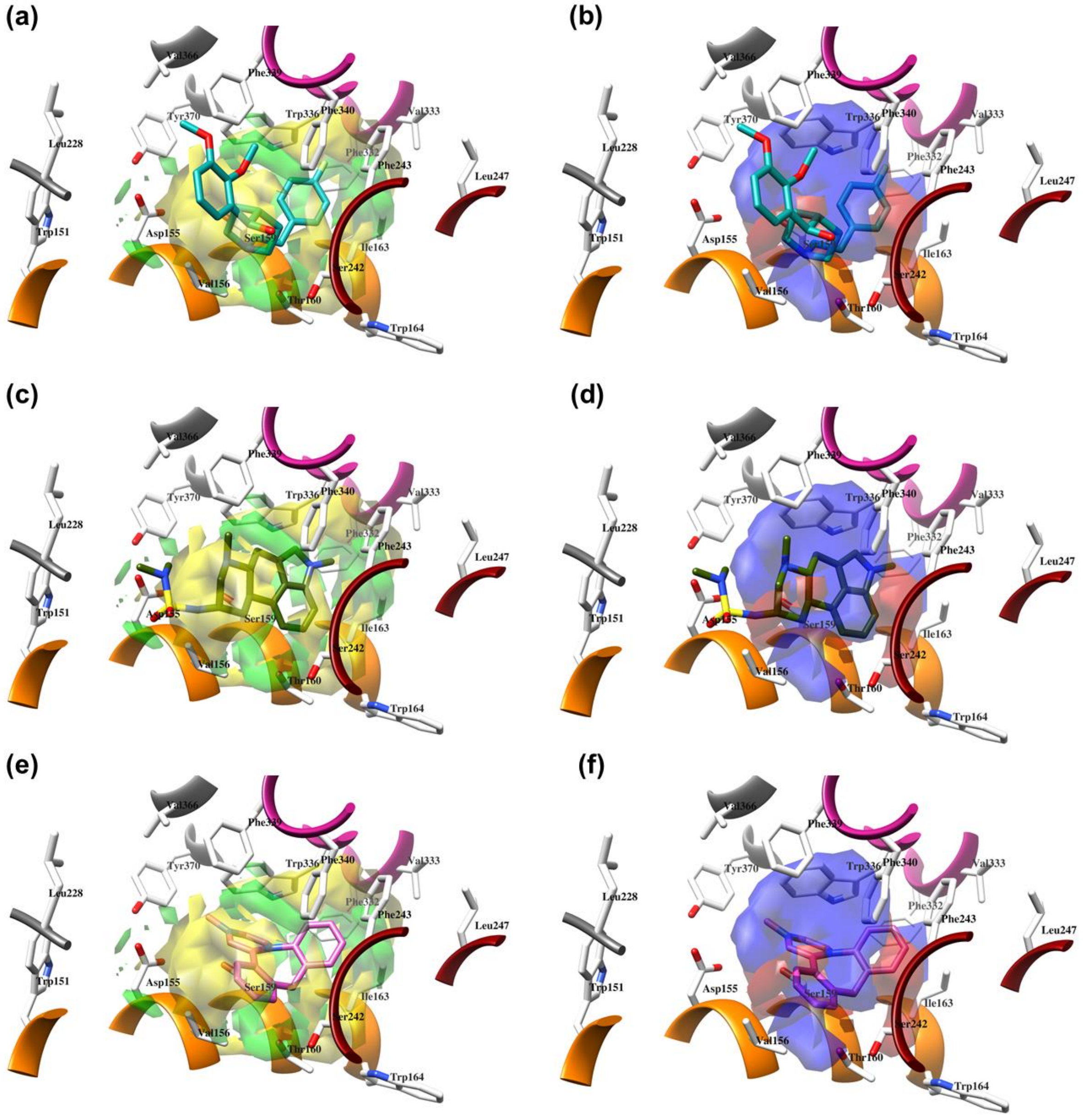

Figure S8. The structure-based alignment within 5-HT$_{2A}$R-DPPC and OH2 probe-based *PLS-coefficients* (positive steric coefficients presented in green, negative steric coefficients depicted in yellow, positive electrostatic coefficients portrayed in red, negative electrostatic coefficients displayed in blue) for **31**(a, b); **32** (c, d); **33** (e, f). For the clarity of presentation, DPPC was omitted.

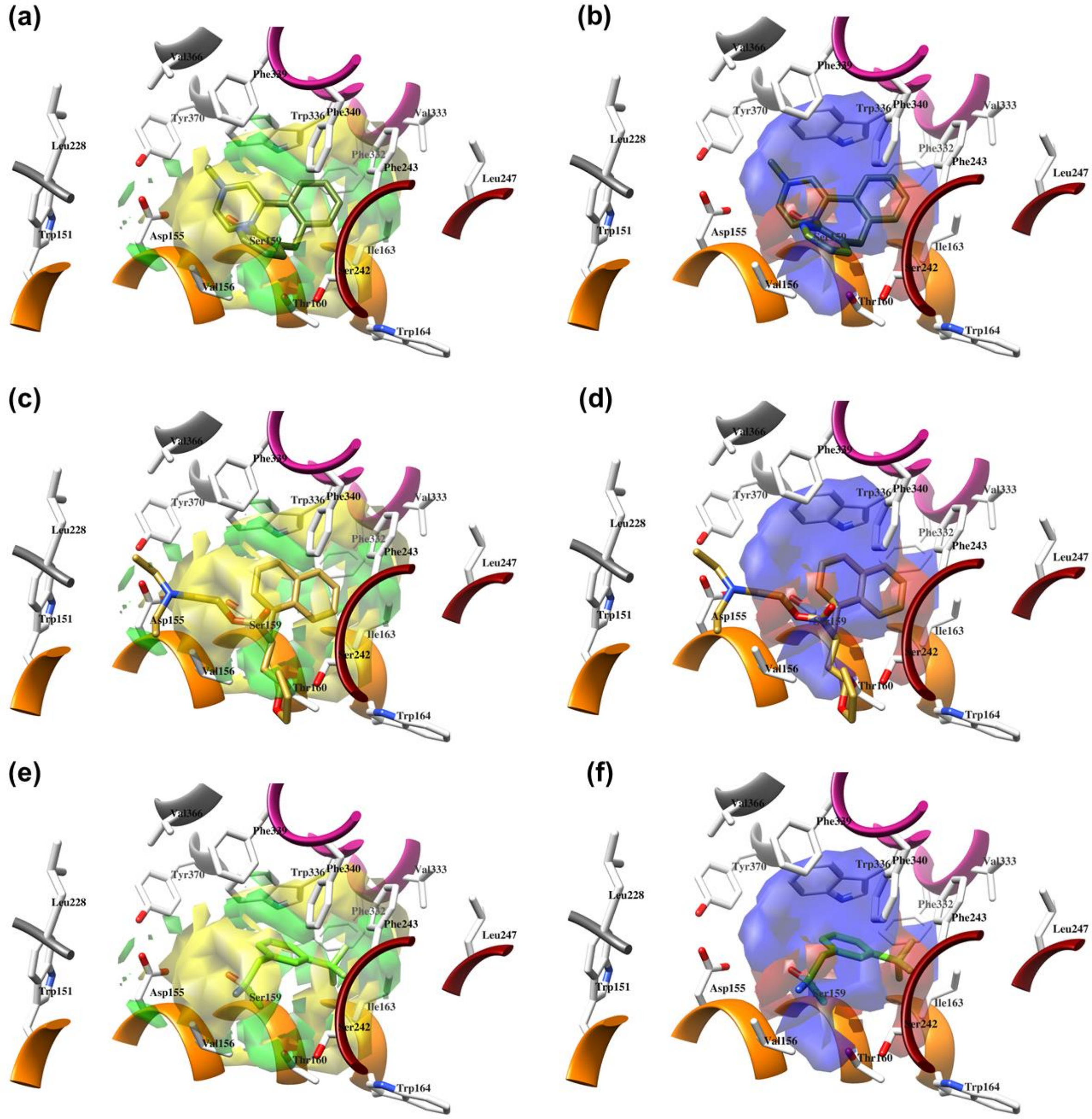

Figure S9. The structure-based alignment within 5-HT$_{2A}$R-DPPC and OH2 probe-based *PLS-coefficients* (positive steric coefficients presented in green, negative steric coefficients depicted in yellow, positive electrostatic coefficients portrayed in red, negative electrostatic coefficients displayed in blue) for **34** (a, b); **35** (c, d); **36** (e, f). For the clarity of presentation, DPPC was omitted.

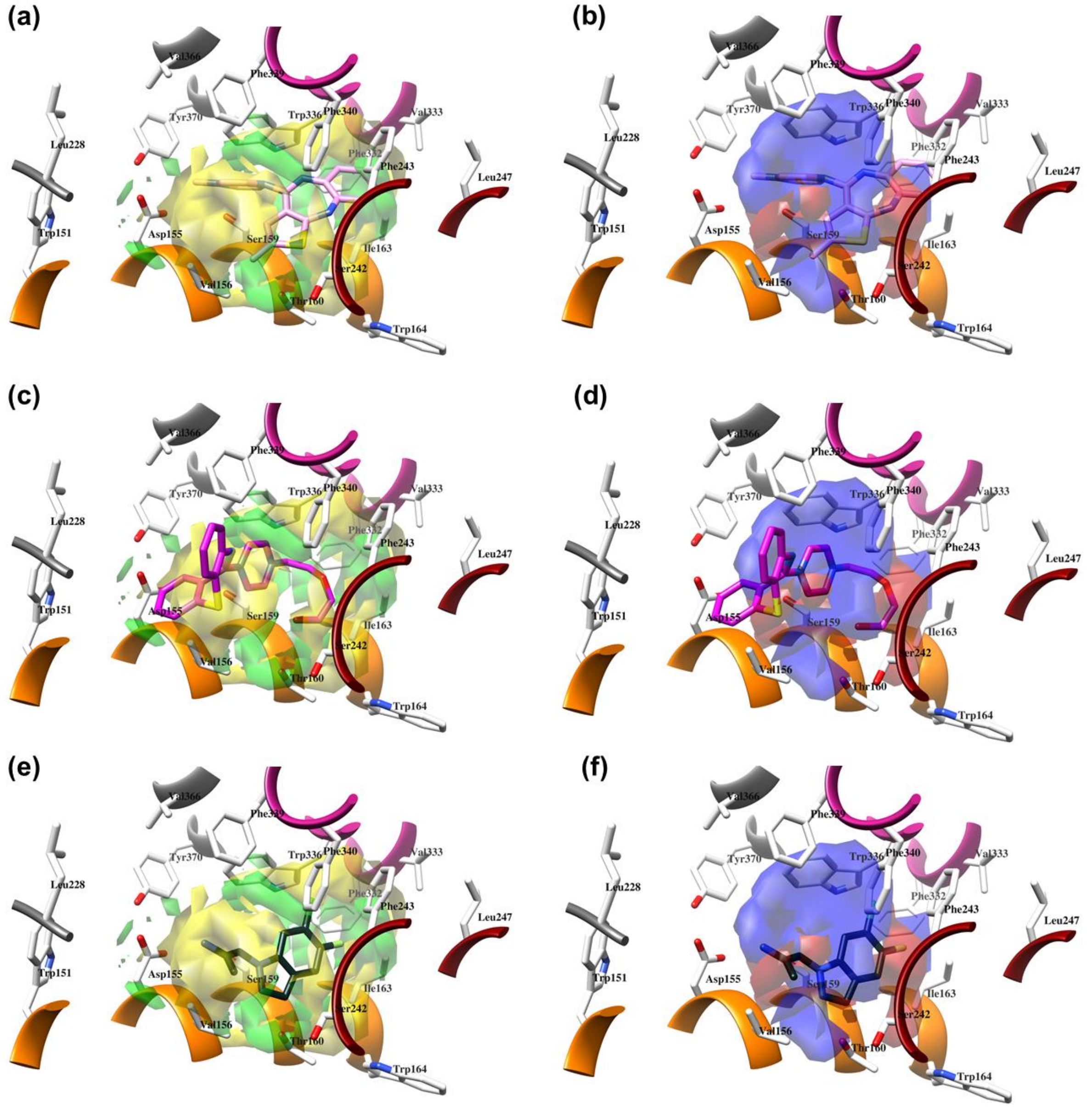

Figure S10. The structure-based alignment within 5-HT_{2A}R-DPPC and OH2 probe-based *PLS-coefficients* (positive steric coefficients presented in green, negative steric coefficients depicted in yellow, positive electrostatic coefficients portrayed in red, negative electrostatic coefficients displayed in blue) for **37** (a, b); **38** (c, d); **39** (e, f). For the clarity of presentation, DPPC was omitted.

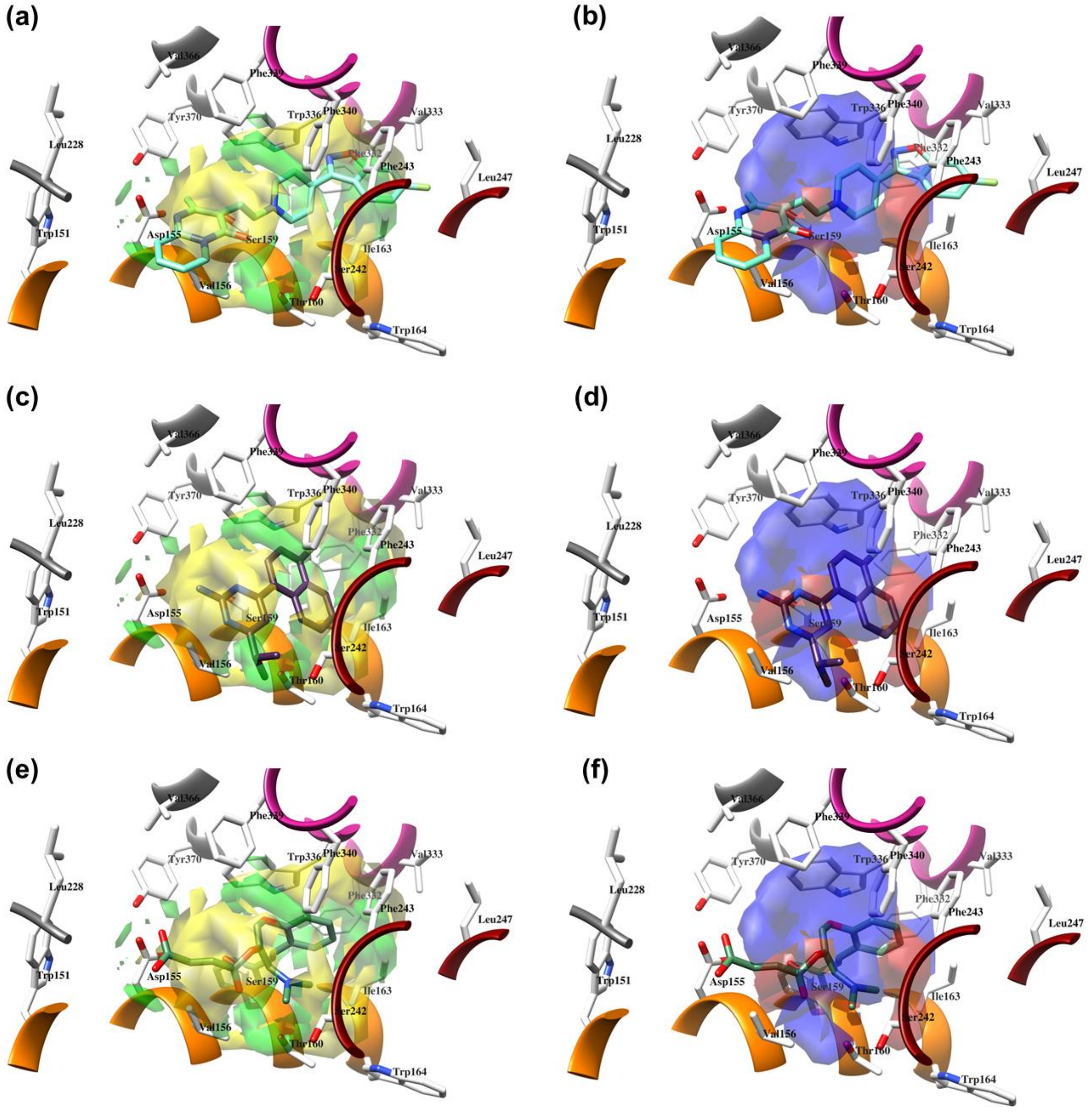

Figure S11. The structure-based alignment within 5-HT$_{2A}$R-DPPC and OH2 probe-based *PLS-coefficients* (positive steric coefficients presented in green, negative steric coefficients depicted in yellow, positive electrostatic coefficients portrayed in red, negative electrostatic coefficients displayed in blue) for **40** (a, b); **41** (c, d); **42** (e, f). For the clarity of presentation, DPPC was omitted.

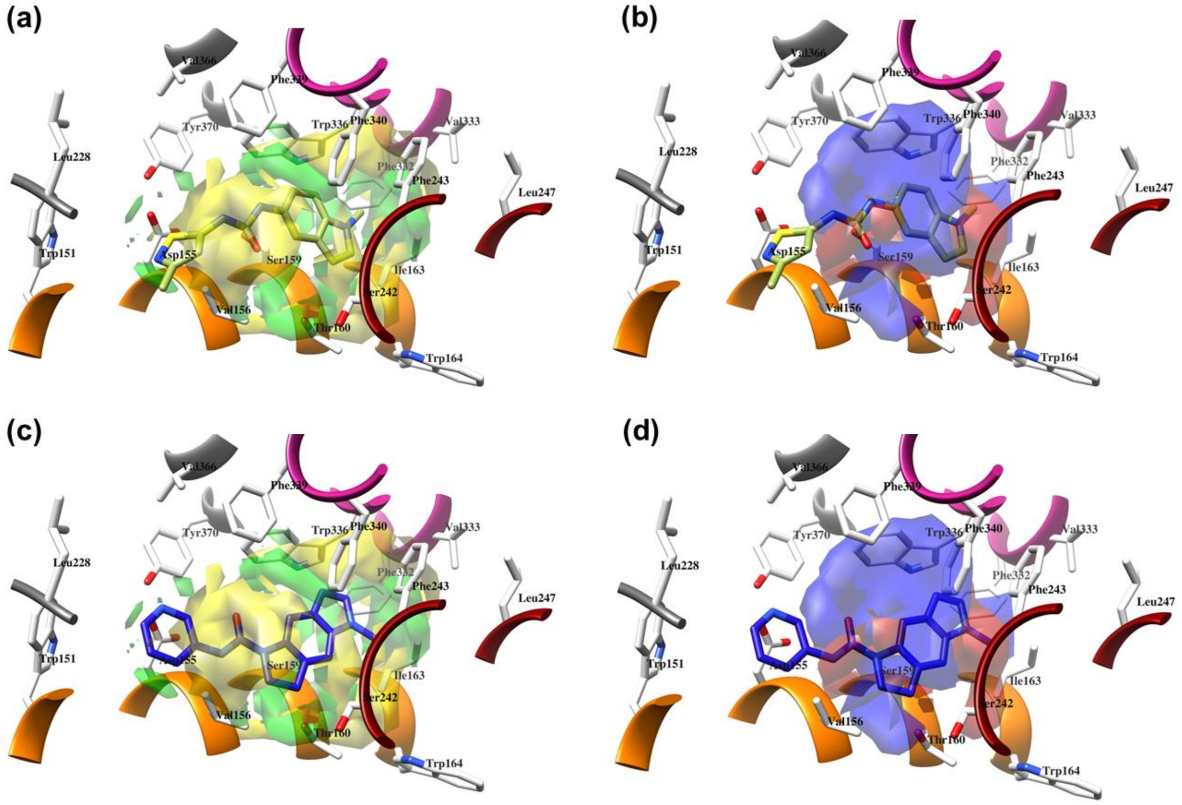

Figure S12. The structure-based alignment within 5-HT2AR-DPPC and OH2 probe-based *PLS-coefficients* (positive steric coefficients presented in green, negative steric coefficients depicted in yellow, positive electrostatic coefficients portrayed in red, negative electrostatic coefficients displayed in blue) for **43** (a, b); **44** (c, d). For the clarity of presentation, DPPC was omitted.

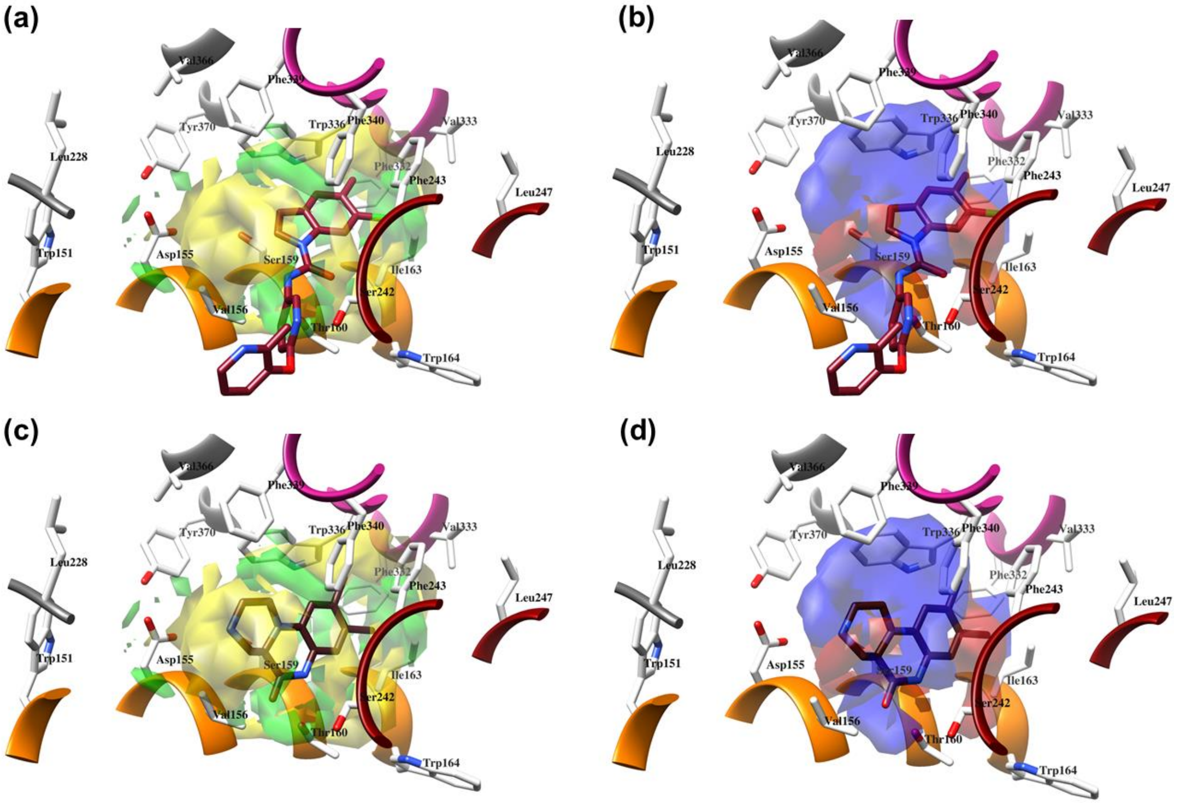

Figure S13. The structure-based alignment within 5-HT$_{2A}$R-DPPC and OH2 probe-based *PLS-coefficients* (positive steric coefficients presented in green, negative steric coefficients depicted in yellow, positive electrostatic coefficients portrayed in red, negative electrostatic coefficients displayed in blue) for **45** (a, b); **46** (c, d). For the clarity of presentation, DPPC was omitted.

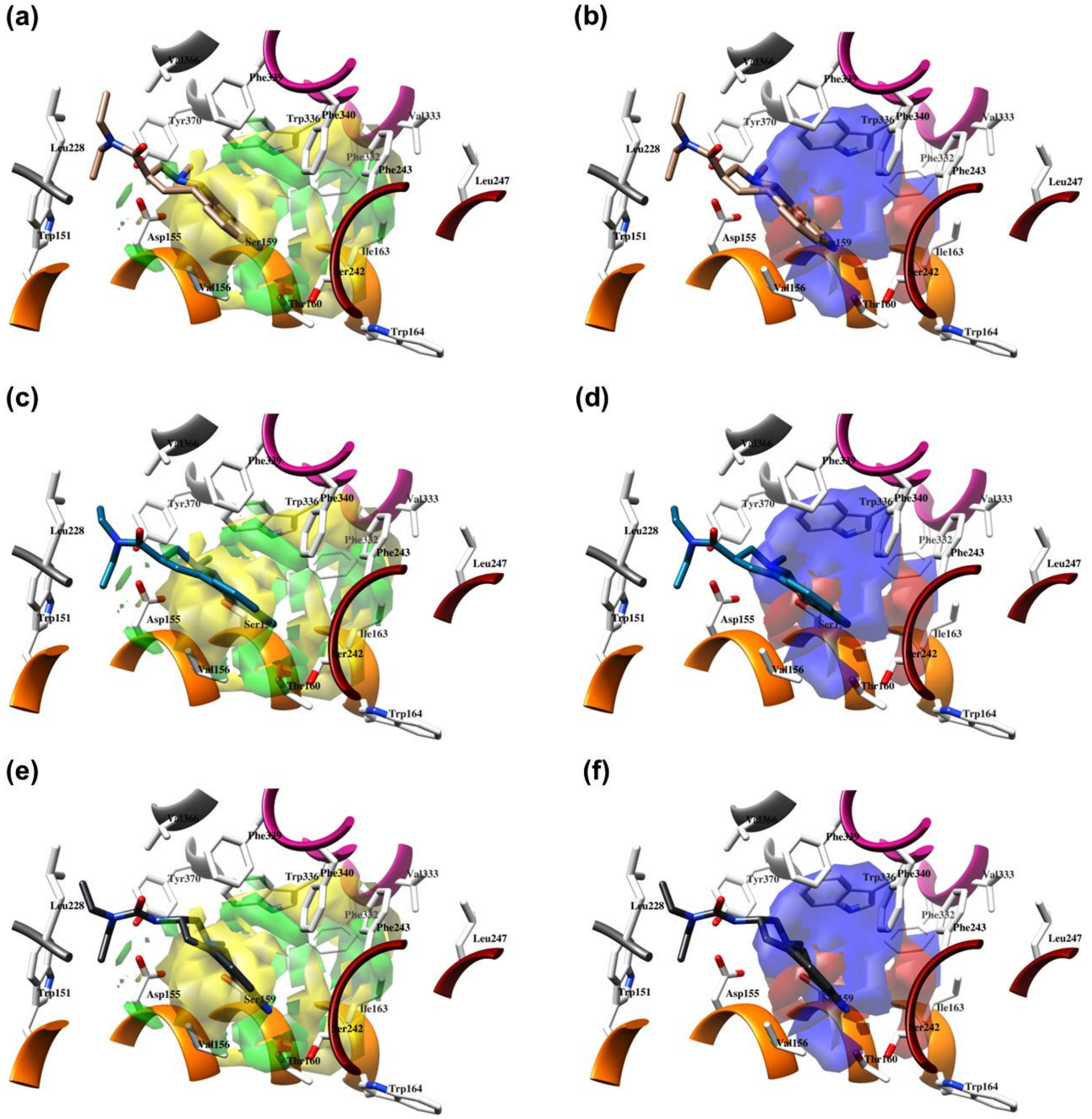

Figure S14. The structure-based alignment within 5-HT$_{2A}$R-DPPC and OH2 probe-based *PLS-coefficients* (positive steric coefficients presented in green, negative steric coefficients depicted in yellow, positive electrostatic coefficients portrayed in red, negative electrostatic coefficients displayed in blue) for **6WGT** (a, b); **7WC6** (c, d); **7WC7** (e, f). For the clarity of presentation, DPPC was omitted.

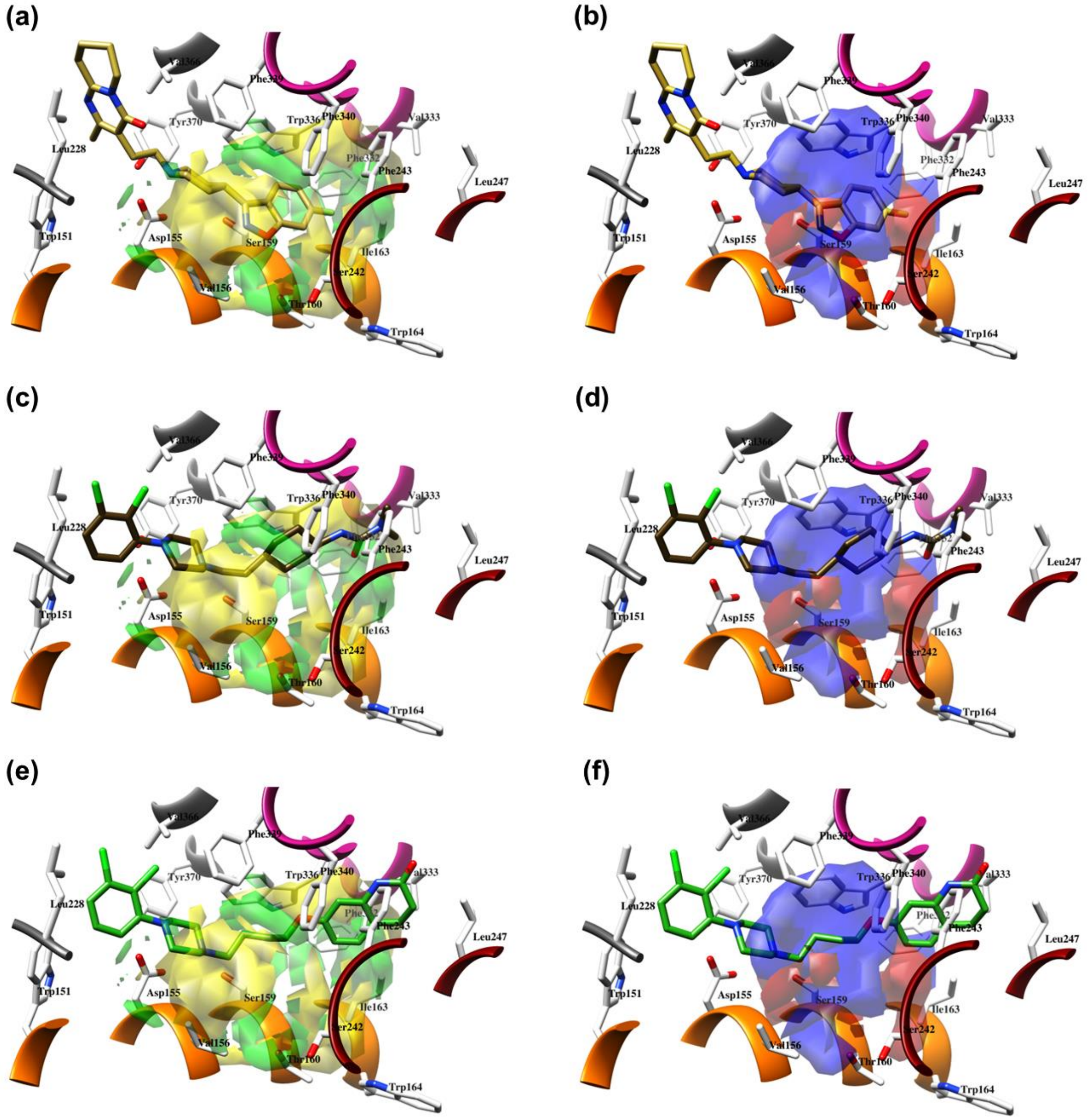

Figure S15. The structure-based alignment within 5-HT2AR-DPPC and OH2 probe-based *PLS-coefficients* (positive steric coefficients presented in green, negative steric coefficients depicted in yellow, positive electrostatic coefficients portrayed in red, negative electrostatic coefficients displayed in blue) for **6A93** (a, b); **7VOD** (c, d); **7VOE** (e, f). For the clarity of presentation, DPPC was omitted.

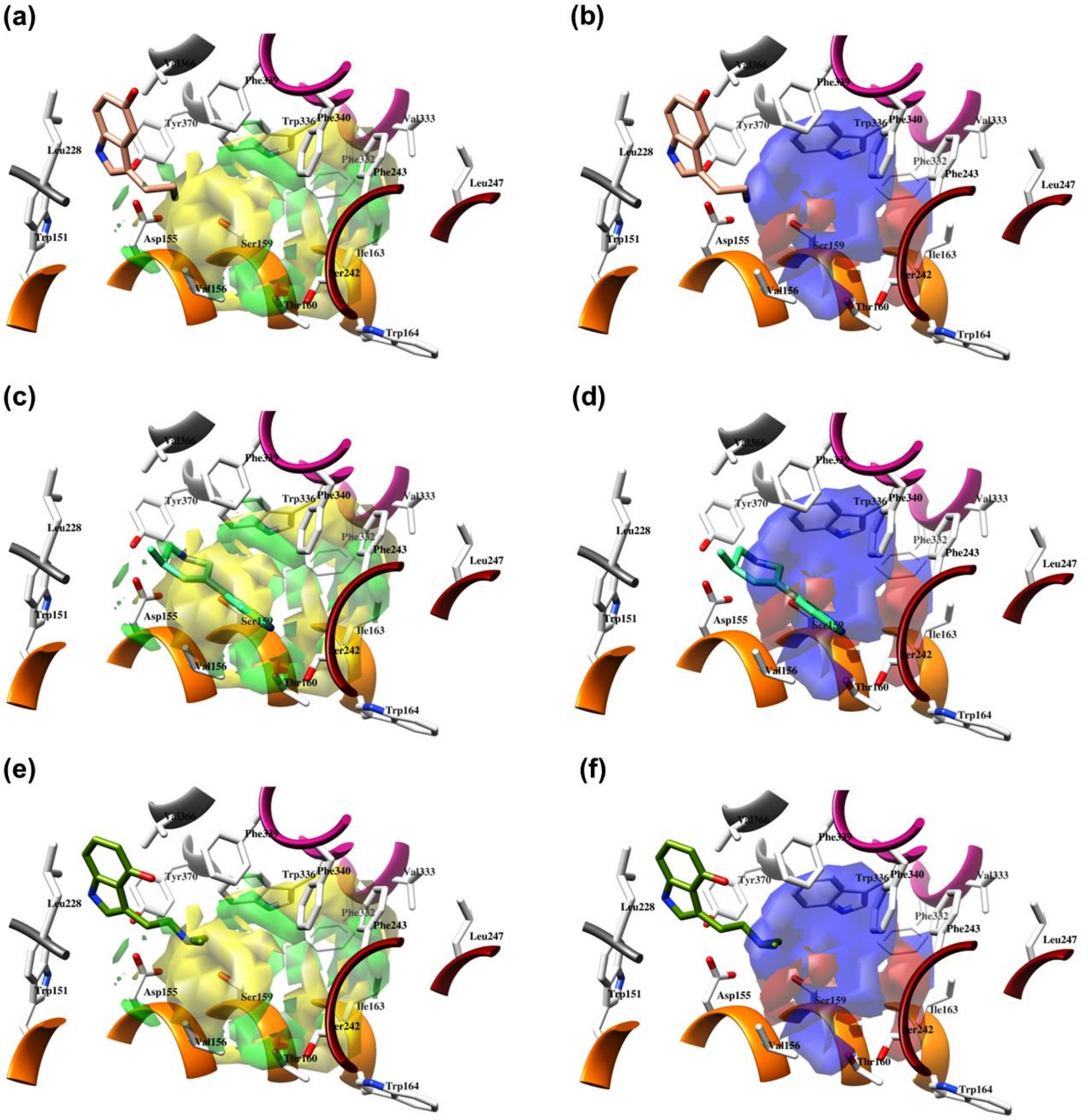

Figure S16. The structure-based alignment within 5-HT$_{2A}$R-DPPC and OH2 probe-based *PLS-coefficients* (positive steric coefficients presented in green, negative steric coefficients depicted in yellow, positive electrostatic coefficients portrayed in red, negative electrostatic coefficients displayed in blue) for **7WC4** (a, b); **7RAN** (c, d); **7WC5** (e, f). For the clarity of presentation, DPPC was omitted.

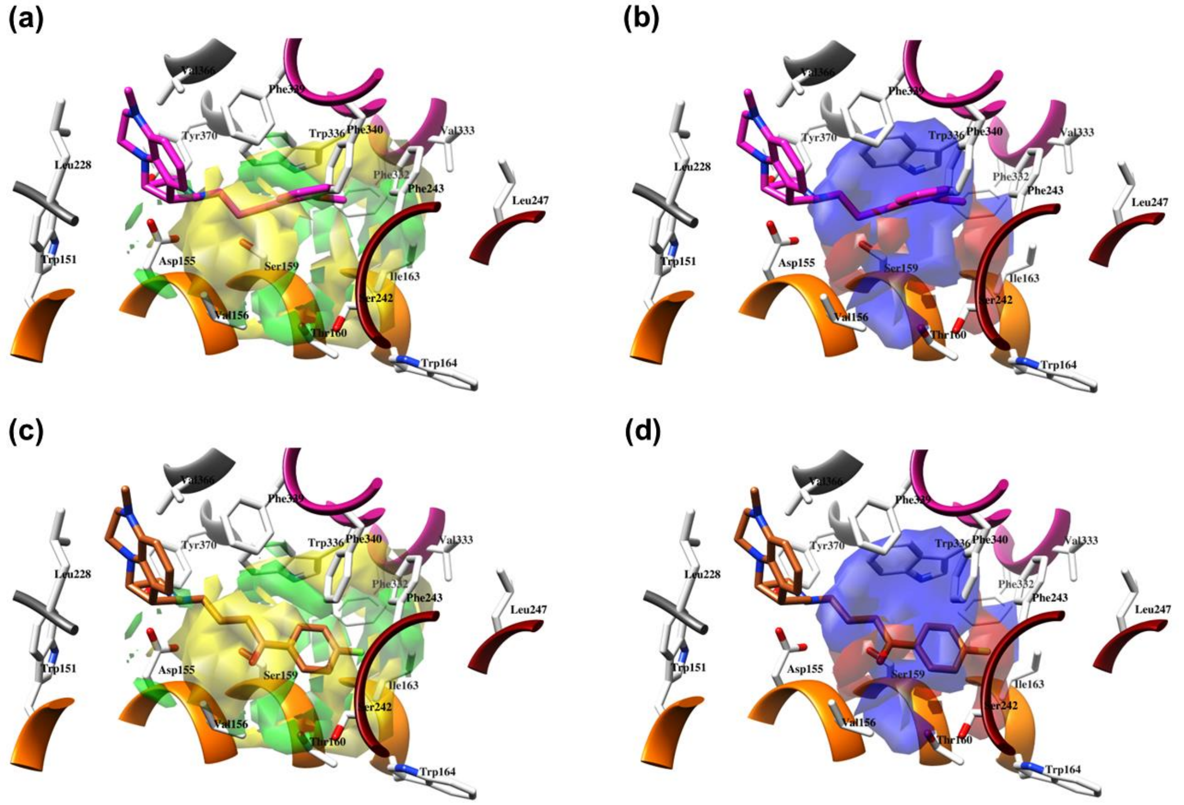

Figure S17. The structure-based alignment within 5-HT$_{2A}$R-DPPC and OH2 probe-based *PLS-coefficients* (positive steric coefficients presented in green, negative steric coefficients depicted in yellow, positive electrostatic coefficients portrayed in red, negative electrostatic coefficients displayed in blue) for **7WC9** (a, b); **7WC8** (c, d). For the clarity of presentation, DPPC was omitted.
